# Clinical Significance of NOTCH1 and NOTCH2 Expression in Gastric Carcinomas: An Immunohistochemical Study

**DOI:** 10.3389/fonc.2015.00094

**Published:** 2015-04-22

**Authors:** Lukas Bauer, Agnes Takacs, Julia Slotta-Huspenina, Rupert Langer, Karen Becker, Alexander Novotny, Katja Ott, Axel Walch, Alexander Hapfelmeier, Gisela Keller

**Affiliations:** ^1^Institute of Pathology, Technische Universität München, Munich, Germany; ^2^Department of Pathology, University of Bern, Bern, Switzerland; ^3^Department of Surgery, Technische Universität München, Munich, Germany; ^4^Department of Surgery, Klinikum Rosenheim, Rosenheim, Germany; ^5^Institute of Pathology, Helmholtz-Zentrum München, Neuherberg, Germany; ^6^Institute of Medical Statistics and Epidemiology, Technische Universität München, Munich, Germany

**Keywords:** stomach neoplasms, receptor NOTCH1, receptor NOTCH2, prognosis, chemotherapy, immunohistochemistry

## Abstract

**Background:**

NOTCH signaling can exert oncogenic or tumor suppressive functions and can contribute to chemotherapy resistance in cancer. In this study, we aimed to clarify the clinicopathological significance and the prognostic and predictive value of NOTCH1 and NOTCH2 expression in gastric cancer (GC).

**Methods:**

NOTCH1 and NOTCH2 expression was determined immunohistochemically in 142 primarily resected GCs using tissue microarrays and in 84 pretherapeutic biopsies from patients treated by neoadjuvant chemotherapy. The results were correlated with survival, response to therapy, and clinico-pathological features.

**Results:**

Primarily resected patients with NOTCH1-negative tumors demonstrated worse survival. High NOTCH1 expression was associated with early-stage tumors and with significantly increased survival in this subgroup. Higher NOTCH2 expression was associated with early-stage and intestinal-type tumors and with better survival in the subgroup of intestinal-type tumors. In pretherapeutic biopsies, higher NOTCH1 and NOTCH2 expression was more frequent in non-responding patients, but these differences were statistically not significant.

**Conclusion:**

Our findings suggested that, in particular, NOTCH1 expression indicated good prognosis in GC. The close relationship of high NOTCH1 and NOTCH2 expression with early tumor stages may indicate a tumor-suppressive role of NOTCH signaling in GC. The role of NOTCH1 and NOTCH2 in neoadjuvantly treated GC is limited.

## Introduction

The NOTCH signaling cascade is a highly conserved signaling pathway, with a crucial role in developmental processes and differentiation programs in various tissues. Aberrant NOTCH signaling has been implicated in carcinogenesis in various organs, and oncogenic, as well as tumor-suppressive functions, have been described (1, 2). In addition, there has been increasing evidence that NOTCH signaling contributes essentially to a drug-resistant phenotype of tumor cells (3).

There are four NOTCH receptors (NOTCH1, 2, 3, 4), which can transmit activating signals from the cell membrane to the nucleus, leading then to the transcription of specific target genes (1, 2). Different and partly opposite roles in one tumor entity have been found for NOTCH1 and NOTCH2, indicating specific functions of the two receptors (4).

In gastric cancer (GC), NOTCH1 and NOTCH2 are believed to contribute to tumor progression (5–7). NOTCH1 has been reported to be a marker of poor prognosis, and increased expression of the NOTCH intracellular domain (NICD) was associated with the presence of lymph node metastasis and worse survival (8, 9).

In a recent study, we demonstrated the prognostic relevance of *NOTCH2* gene expression in residual tumor cells after chemotherapy of neoadjuvantly treated GC patients. In addition, an increase in *NOTCH2* expression in the residual tumor cells compared to the pretherapeutic biopsies was shown, indicating a prominent role of *NOTCH2* in chemotherapy resistance in GC (10).

In this study, we first aimed to clarify the role of NOTCH1 and NOTCH2 protein expression in gastric carcinomas relative to prognosis and to the clinico-pathological characteristics of patients not treated by chemotherapy. Second, we aimed to determine the predictive and prognostic relevance of both proteins for neoadjuvant chemotherapy, and we included a group of pretherapeutic tumor biopsies of neoadjuvantly treated GC patients in the study.

## Materials and Methods

### Patients and specimens

Gastric carcinomas from 226 patients operated on in the Department of Surgery at the Technische Universität München, Germany, between 1992 and 2010 were analyzed. The study population encompassed two subgroups and the patients’ characteristics are shown in Table 1.

**Table 1:** **Patient characteristics**.

Variable	Category	Primary resected tumors *n* (%)	Pretherapeutic biopsies *n* (%)
Patients		142 (100)	84 (100)
Age (years)	Median	70.9	63.4
	Range	39.5–99.9	39.2–77.6
Sex	Female	55 (39)	23 (27)
	Male	87 (61)	61 (73)
Tumor localisation^a^	Proximal	33 (23)	59 (70)
	Medial	39 (27)	14 (17)
	Distal	54 (38)	10 (12)
	Total	12 (8)	1 (1)
Laurén classification	Intestinal	76 (54)	28 (33)
	Non-intestinal	66 (46)	56 (67)
Tumor grade	G1 + 2	26 (18)	9 (11)
	G3 + 4	116 (82)	75 (89)
Resection category^b^	R0	82 (58)	68 (81)^d^
	R1 + 2	54 (38)	15 (18)^d^
pT category^c^	pT1 + 2	64 (45)	63 (75)^d^,^e^
	pT3 + 4	78 (55)	20 (24)^d^,^e^
pN category^c^	pN0	36 (25)	36 (43)^d^,^f^
	pN1 + 2 + 3	106 (75)	47 (56)^d^,^f^

*^a^For four patients, the tumor localization was unknown*.

*^b^For six patients, the resection category was unknown*.

*^c^TNM Classification of Malignant Tumors, 6th Edition, UICC (doi: 10.1371/journal.pone.0044566.t001)*.

*^d^One patient with progressive disease was not submitted to surgery*.

*^e^Corresponds to ypT*.

*^f^Corresponds to ypN*.

The first subgroup consisted of 142 resected specimens of patients who had been treated by surgical resection without neoadjuvant chemotherapy. Follow-up was calculated from the day of surgery until the last contact with the patient. Patients who died within 4 weeks after surgery were excluded from survival analysis. Overall survival (OS) was defined from the day of surgery to death from any cause. Survival data were available for 126 of the 142 patients. The median follow-up time for these patients was 40.1 months (range: 0.3–116.1 months), and the median OS was 15.8 months (95% CI 8.1–23.5 months).

The second subgroup encompassed 84 pretherapeutic biopsies of advanced gastric carcinoma patients (cT3 + cT4 only), who were treated with a platinum/5FU-based neoadjuvant chemotherapy, as reported previously (10).

Response to therapy was evaluated histopathologically, as described previously (11). In brief, all patients with <10% residual tumor cells per tumor bed [tumor regression grade 1 (TRG1)] were classified as responders. All other patients with 10–50% (TRG2) and with more than 50% residual tumor cells per tumor bed (TRG3), as well as patients who demonstrated tumor progression during chemotherapy, were classified as non-responders. Among the 84 patients, there were 22 (26%) responding and 62 (74%) non-responding patients.

Follow-up for these patients was calculated from the first day of chemotherapy until the last contact with the patient. OS was defined from the first day of chemotherapy until death from any cause. The median follow-up time for these patients was 40.6 months (range: 7.0–84.7 months), and the median OS was 50.6 months (mean OS 52.8 months 95% CI: 50.0–60.7). The responders had significantly better OS than the non-responders (*p*_log-rank_ = 0.041; median OS not reached and 50.6 months, respectively; mean OS 63.9 months, 95% CI: 50.0–77.7 versus 44.3 months, 95% CI 36.5–52.2, respectively).

The use of the tissue samples was approved by the local ethics committee of the Faculty of Medicine, Technische Universität München, Germany (Ref. 4071/11, 21/04/2011) and the informed consent of the patients was obtained according to the institutional regulations.

### Immunohistochemistry

Immunohistochemistry was performed on formalin-fixed paraffin embedded (FFPE) tumor biopsies and on tissue microarrays (TMAs) of resected specimens. Tissue cores with a diameter of 1.0 mm from randomly selected tumor areas were used for the preparation of the TMAs. The monoclonal NOTCH1 (bTAN20-s) and NOTCH2 antibodies (C651.6DbHN) were obtained from the Developmental Studies Hybridoma Bank (DSHB, the University of Iowa, Department of Biology, Iowa City, IA, USA) and were used undiluted for NOTCH1 and at a dilution of 1:30 for NOTCH2. Specificity of the antibodies was determined by western blotting, using the GC cell line MKN28 with short hairpin RNA-mediated knockdown of *NOTCH1* or *NOTCH2* expression, respectively (Figure S1 in Supplementary Material).

Expression was evaluated by two researchers (AT and RL for NOTCH2, LB and JSH for NOTCH1). Cytoplasmic and nuclear staining was determined separately. Negative, weak, medium, and strong staining intensities were scored as 0, 1, 2, and 3, respectively. The percentage of tumor cells with stained cytoplasm/nucleus was scored as 0 (negative), 1 (<10%), 2 (10 to <50%), 3 (50 to <80%), or 4 (≥80%). Multiplication of staining intensities and percentages resulted in a staining index (SI) ranging from 0 to 12, as described previously (12). We defined an SI of 0 as negative expression, 1–2 as weak, 3–6 as moderate, and 8–12 as strong expression.

### Statistical analysis

Associations between the SIs and qualitative clinical and histopathological features were tested by the χ^2^ test or Fisher’s exact test, depending on the cell counts of the corresponding contingency tables. Survival was estimated by Kaplan–Meier curves. The minimal *p*-value of log-rank tests was used to dichotomize the patients according to their various expression groups. Corresponding *p*-values served as effect measurements to determine optimality and should not interpreted in a probabilistic manner. For that purpose, appropriate *p*-values of maximally selected log-rank statistics were determined in a permutation test framework and are indicated as *p*_max_. In multivariate Cox regression analysis, stepwise forward variable selection was performed based on likelihood ratio tests. Because our study was an explorative study, there was no adjustment for multiple testing. All of the statistical testing was performed using a two-sided 5% significance level. SPSS software (IBM, Armonk, NY, USA), version 22.0, was used.

## Results

### Frequency of NOTCH1 and NOTCH2 expression in primarily resected gastric carcinomas

NOTCH1 expression was evaluable in 133 of the 142 primarily resected tumor specimens. Strong cytoplasmic expression was found in 10 (8%) of the 133 tumors, 38 (29%) demonstrated moderate expression, 42 (32%) demonstrated weak expression, and 43 (32%) were negative.

NOTCH2 expression was evaluable in all of the 142 primarily resected tumors. Nine tumors (6%) showed strong cytoplasmic expression, 61 (43%) showed moderate cytoplasmic expression, 53 (37%) showed weak cytoplasmic expression, and 19 (13%) were negative.

None of the tumors showed nuclear NOTCH1 expression. Regarding nuclear NOTCH2 expression, 64 (45%) of the 142 tumors were negative, 63 (44%) demonstrated weak expression, and 15 (11%) tumors showed moderate/strong expression.

**Figure 1:**
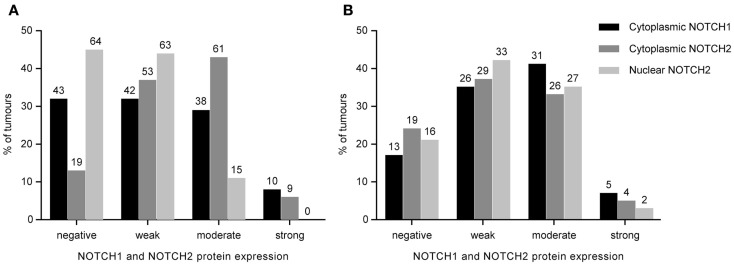
**Distribution of NOTCH1 and NOTCH2 protein expression in 142 primarily resected gastric carcinomas (A) and 84 pretherapeutic biopsies of neoadjuvantly treated patients (B)**. Staining indices are grouped into negative, weak, moderate, and strong expression categories as described in the Section “Materials and Methods.” The absolute number of cases is indicated above the bars.

**Figure 2:**
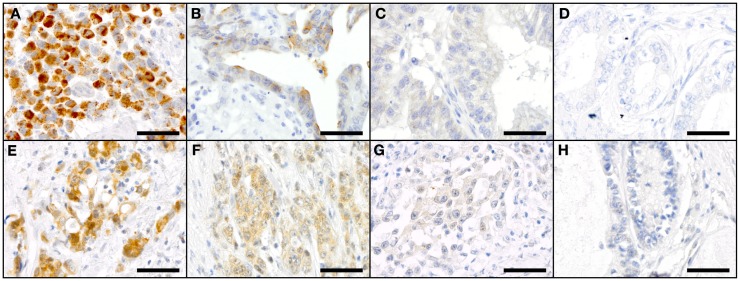
**Staining patterns for NOTCH1 (A–D) and NOTCH2 (E–H) in primary resected GC specimen**. NOTCH1: **(A)** high-intensity cytoplasmic staining (SI 12), **(B)** medium-intensity cytoplasmic staining (SI 4), **(C)** low-intensity cytoplasmic staining (SI 2), and **(D)** negative cytoplasmic staining for NOTCH1 (SI 0); NOTCH2: **(E)** high-intensity cytoplasmic staining (SI 9) and low-intensity nuclear staining (SI 1), **(F)** medium-intensity cytoplasmic staining (SI 6) and low-intensity nuclear staining (SI 1); **(G)** low-intensity cytoplasmic staining (SI 3) and negative nuclear staining (SI 0); and **(H)** negative cytoplasmic and nuclear staining for NOTCH2. Scale bars: 50 μm.

The expression frequencies for primary resected tumors are shown in Figure 1A. Examples of staining patterns are shown in Figure 2.

### Expression of NOTCH1 and NOTCH2 in primarily resected gastric carcinomas and correlation with clinico-pathological characteristics

Expression was analyzed for an association with the clinico-pathological characteristics and with the survival of the patients in the subgroup of 142 primarily resected carcinomas. NOTCH1 was evaluable in 133 of the 142 cases and NOTCH2 in all 142 cases.

**Figure 3:**
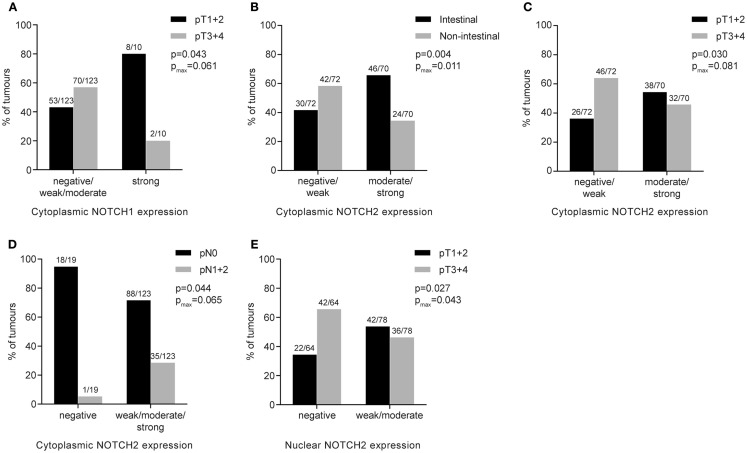
**Association of NOTCH1 or NOTCH2 protein expression with clinico-pathological features of primary resected gastric carcinomas**. Numbers above the bars indicate the absolute number of cases. *p*-Values were determined by the χ^2^-test or Fisher’s exact test; *p*_max_ corresponds to *p*-values for maximally selected statistics. **(A)** Cytoplasmic NOTCH1 expression and association with tumor stage; **(B)** cytoplasmic NOTCH2 expression and association with histopathological tumor type; **(C)** cytoplasmic NOTCH2 expression and association with tumor stage; **(D)** cytoplasmic NOTCH2 expression and association with lymph node status; **(E)** nuclear NOTCH2 expression and association with tumor stage.

Strong NOTCH1 expression was associated with earlier tumor stages (*p* = 0.043; *p*_max_ = 0.061) (Figure 3A). No significant association of NOTCH1 expression with histopathological type according to Laurén, tumor differentiation, lymph node status, tumor location, or age was found.

Regarding NOTCH2, higher cytoplasmic expression was significantly associated with intestinal-type tumors (*p* = 0.004; *p*_max_ = 0.011) (Figure 3B), was more prevalent in lower tumor stages (pT1 + pT2) (*p* = 0.030; *p*_max_ = 0.081) (Figure 3C) and was more frequently found in patients with negative lymph node status (*p* = 0.044; *p*_max_ = 0.065) (Figure 3D). Regarding nuclear NOTCH2 expression, positive expression (weak/moderate/strong) was associated with lower tumor stages (pT1 + pT2), compared to advanced tumor stages (pT3 + pT4) (*p* = 0.027; *p*_max_ = 0.043) (Figure 3E). No other significant differences were found.

### Expression of NOTCH1 and NOTCH2 in primarily resected gastric carcinomas and prognosis

**Figure 4:**
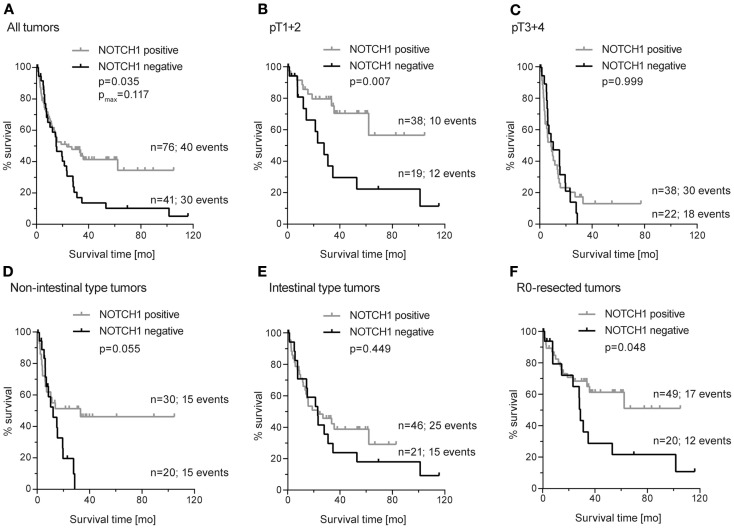
**Kaplan–Meier survival curves for NOTCH1 protein expression in primary resected gastric carcinomas**. **(A)** All tumors (median OS: 15.1 months; 95% CI 8.3–22.0 versus 23.3 months; 95% CI 4.6–26.1); **(B)** subgroup of early-stage tumors (pT1 + 2) (median OS: 28.0 months; 95% CI 16.2–40.0 versus median OS not reached); **(C)** subgroup of advanced-stage tumors (pT3 + 4) (median OS: 10.3 months; 95% CI 0–21.8 versus 8.7 months; 95% CI 3.1–14.3); **(D)** subgroup of non-intestinal-type tumors (median OS: 12.2 months; 95% CI 4.1–20.2 versus 33.2 months; 95% CI not applicable); **(E)** subgroup of intestinal-type tumors (median OS: 23.1 months; 95% CI 12.0–34.2 versus 23.3 months; 95% CI 3.0–43.6); **(F)** subgroup of patients with completely resected tumors (R0) (median OS: 28.8 months; 95% CI 27.1–30.5 versus median OS not reached); *p*-values were determined by log-rank statistics; *p*_max._ corresponds to *p*-values for maximally selected statistics.

Patients who did not show any NOTCH1 expression in their tumors demonstrated significantly worse survival, compared to all other patients having weak, moderate, or strong NOTCH1 expression (*p*_log-rank_ = 0.035; *p*_max_ = 0.117) (Figure 4A).

Analyzing the patients with NOTCH1-negative tumors for an association with survival in the subgroups of (a) early (pT1 + pT2) and advanced (pT3 + pT4) tumor stages separately, (b) in intestinal and non-intestinal-type tumors, and (c) in completely resected (R0) tumors only, revealed significant associations with NOTCH1-negative tumors and worse survival in patients with early tumor stages but not in patients with advanced tumors (*p*_log-rank_ = 0.007 and *p*_log-rank_ = 0.999, respectively) (Figures 4B,C). A trend toward an association of these patients with worse survival was observed in non-intestinal tumors but not in intestinal tumors (*p*_log-rank_ = 0.055 and *p*_log-rank_ = 0.449, respectively) (Figures 4D,E). In addition, patients with NOTCH1-negative tumors showed a significant decrease in survival in the subgroup of patients with completely resected tumors (*p*_log-rank_ = 0.048) (Figure 4F).

Multivariate Cox regression analysis, including NOTCH1 expression and the standard prognostic factors, namely pT, pN, and R-status, revealed no prognostic relevance for NOTCH1 expression neither in the whole study group nor in the subgroup of completely resected patients. In the subgroup of patients with early tumor stages (*n* = 64), NOTCH1 expression was the second most important factor (HR: 0.82, 95% CI: 0.67–0.99, *p* = 0.038) after lymph node metastasis (HR: 3.35, 95% CI 1.30–8.68, *p* = 0.013).

**Figure 5:**
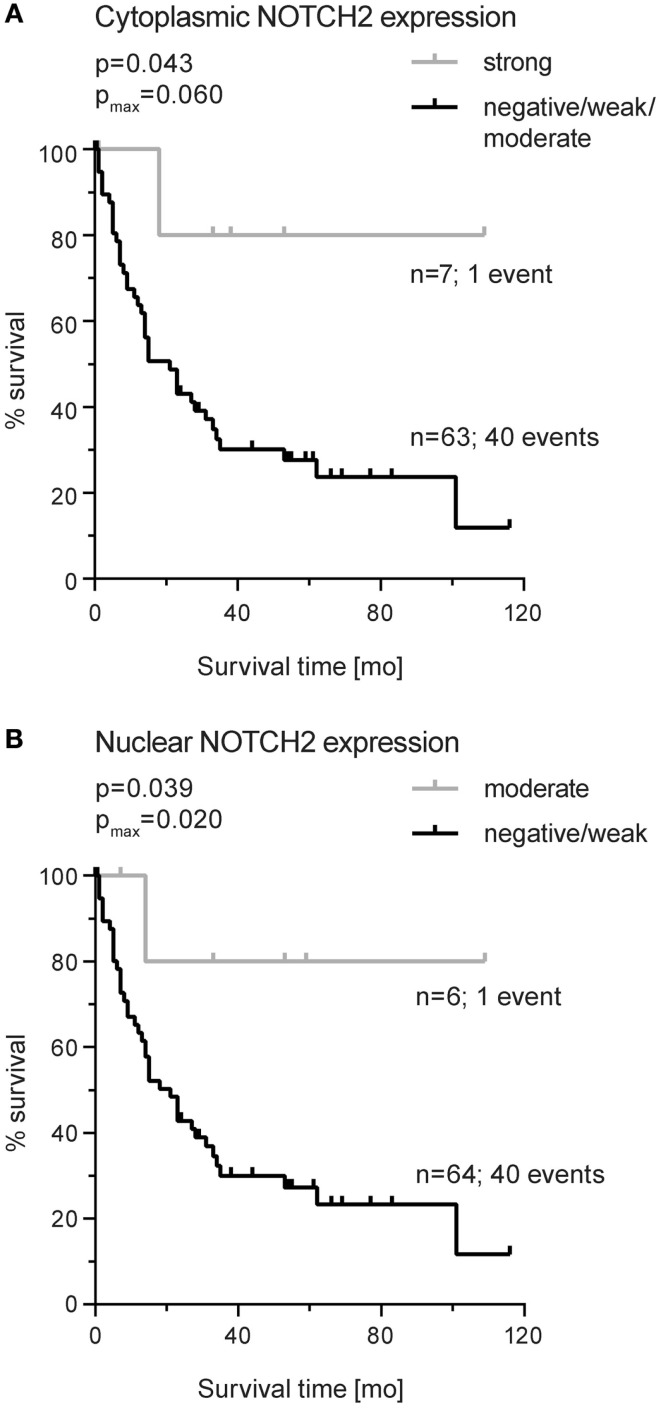
**Kaplan–Meier survival curves for NOTCH2 protein expression in primary resected gastric carcinomas of the intestinal type**. **(A)** Cytoplasmic expression (median OS: not reached versus 21.2 months; 95% CI 11.4–31.3) and **(B)** nuclear expression (median OS: not reached versus 21.4 months; 95% CI 11.6–31.1). *p*-Values were determined by log-rank statistics; *p*_max_ corresponds to *p*-value for maximally selected statistics.

Considering NOTCH2, cytoplasmic or nuclear expression did not demonstrate an association with survival. Subgroup analyses showed an association of strong cytoplasmic NOTCH2 expression with increased survival in patients with intestinal-type tumors (*p*_log-rank_ = 0.043; *p*_max_ = 0.060) (Figure 5A). In addition, moderate/high nuclear NOTCH2 expression was also significantly associated with better survival in this subgroup (*p*_log-rang_ = 0.039; *p*_max_ = 0.020) (Figure 5B). Multivariate analysis in intestinal tumors indicated R-status (HR: 2.78, 95% CI: 1.39–5.55, *p* = 0.004) and tumor stage (HR: 2.61, 95% CI: 1.28–5.30, *p* = 0.008) as independent prognostic factor but not NOTCH2 expression.

### Frequency of NOTCH1 and NOTCH2 expression in pretherapeutic biopsies of neoadjuvantly treated patients

Expression of NOTCH1 was evaluable in 75 of 84 pretherapeutic biopsies of patients treated with a neoadjuvant chemotherapy. Thirteen (17%) of the 75 samples were negative for NOTCH1 protein expression, 26 (35%) showed a weak cytoplasmic expression, 31 (41%) showed a moderate expression, and 5 (7%) demonstrated a strong cytoplasmic NOTCH1 expression. None of the samples showed nuclear NOTCH1 expression.

NOTCH2 was evaluable in 78 of the 84 samples. Cytoplamic NOTCH2 expression was strong in 4 (5%), moderate in 26 (33%), weak in 29 (37%), and negative in 19 (24%) of these samples. Nuclear NOTCH2 expression was observed to be strong in 2 (3%), moderate in 27 (35%), weak in 33 (42%), and negative in 16 (21%) of pretherapeutic biopsies. The expression frequencies for pretherapeutic biopsies are shown in Figure 1B.

### Expression of NOTCH1 and NOTCH2 in pretherapeutic biopsies and response to therapy

No association of NOTCH1 or NOTCH2 expression with response was found using the classification system of negative (SI 0), weak (SI 1–2), moderate (SI 3–6), and strong (SI 8–12) expression. Searching for an optimal cut-off value to differentiate responding and non-responding patients revealed a trend toward an association of tumors with higher cytoplasmic NOTCH1 (SI ≥ 6) or NOTCH2 (SI ≥ 4) expression with worse response (*p* = 0.056 and *p* = 0.079, respectively) (Table 2). No significant associations with survival were found.

**Table 2:** **NOTCH1 and NOTCH2 protein expression in pretherapeutic tumor biopsies and response to neoadjuvant chemotherapy**.

	NOTCH1			NOTCH2		
	*n* SI^a^ < 4	*n* SI ≥ 4	*p*-value^b^	*n* SI < 3	*n* SI ≥ 3	*p*-value^b^
Responders	18	1	0.056	19	2	0.079
Non-responders	41	15		40	17	
Total	59	16		59	19	

*^a^Staining index*.

*^b^Fisher’s exact test*.

## Discussion

In this study, we addressed the potential clinical impact of NOTCH1 and NOTCH2 expression in GC by analyzing both proteins in a group of patients treated directly by primary resection of their tumors and in another group treated with platinum/5FU-based neoadjuvant chemotherapy.

One of the most interesting results of our study was the finding of higher expression of cytoplasmic NOTCH1 and of cytoplasmic and nuclear NOTCH2 in early tumor stages (pT1 + pT2), compared to advanced stages (pT3 + pT4). This finding indicated that a decrease in expression of both proteins occurred during tumor progression, suggesting a tumor-suppressive function of NOTCH signaling in gastric carcinogenesis. Furthermore, our study showed the prognostic relevance of NOTCH1, with higher expression being associated with increased survival and no lymph node metastasis. Beyond this, an impressive prognostic role for NOTCH1 was found for patients with T1/T2 tumors but not for patients with advanced pT3/pT4 tumors. Patients with higher NOTCH1-expressing tumors in the subgroup with early pathological tumor stages had significantly better survival than those with a low expression, and multivariate analysis indicated that NOTCH1 expression was an independent prognostic factor in this subgroup. This finding characterized NOTCH1 expression as a prognostic factor, particularly during early tumor development, and it additionally supported a possible tumor-suppressive role for NOTCH signaling.

It is well known that NOTCH signaling can exert oncogenic or tumor-suppressive functions and that individual NOTCH receptors can exert opposite functions in context- and time-dependent manners, even in the same tumor type (2). For example, a tumor-suppressive function has been reported for skin and liver cancers, an oncogenic role for NSCLC (2, 13) and a dual role in the same tumor type for colorectal and pancreatic cancers (4, 14). The findings of our study ascribed a tumor-suppressive role to NOTCH signaling in GC. This result was essentially in agreement with a functional analysis of NOTCH2 in a GC cell line, which demonstrated that downregulation of NOTCH2 by siRNA enhanced tumor cell invasion (15). However, our results were in contrast to findings from other studies mainly performed in Asian countries, which have reported a contribution of higher expression of NOTCH1 or NOTCH2 to tumor progression and, in particular, an association of higher expression of NOTCH1 with worse prognosis (5, 7–9, 16). Furthermore, higher NOTCH2 expression was more frequently found in intestinal-type tumors in our study, again inconsistently with an analysis of Asian patients reporting no associations of NOTCH2 expression with histopathological type (6). The reasons for these discrepancies are not clear, but they could be related to differences in the study populations, in the analytical technologies or in the applied scoring methods. In addition, differences related to the specificity of the antibodies might exist. In this context, we would like to emphasize that we demonstrated the specificity of the antibodies that we used in our study by western blotting. In addition, based on the results in the respective *NOTCH1*- and *NOTCH2*-knockdown cells, we showed that cross-reaction of both antibodies was very unlikely. Interestingly, we observed positive nuclear staining only for NOTCH2 and not for NOTCH1. This finding might reflect varying degradation rates of nuclear NOTCH1 and NOTCH2 proteins or variations in the sensitivities of the specific antibodies.

In the subgroup of neoadjuvantly treated GC patients, we identified only a trend toward an association of a higher NOTCH1 and NOTCH2 expression in the pretherapeutic biopsies and worse tumor regression after chemotherapy. Recent studies have indicated an association between NOTCH1 or NOTCH3 and cisplatin resistance in squamous head and neck and ovarian carcinomas and in EBV-associated naso-pharynx carcinomas, respectively (17–19).

We are aware that our study had limitations, mainly related to its retrospective nature and to the limited number of tumors we analyzed. Thus, further studies are needed to delineate in greater detail the role of NOTCH1 and NOTCH2 expression in gastric carcinogenesis and the impact of these molecules in the prediction of response to neoadjuvant chemotherapy in GC. Furthermore, functional analyses are needed to clarify a possible tumor-suppressive role of NOTCH signaling in GC.

Nevertheless, in conclusion and to the best of our knowledge, our study showed for the first time a close relationship of a decrease of NOTCH1 and NOTCH2 expression with tumor progression in GC, and our findings suggested that, in particular, NOTCH1 expression was a marker for good prognosis. These findings taken together may suggest a tumor-suppressive role of the NOTCH signaling pathway in GC.

## Author Contributions

Study design and writing of the manuscript (LB and GK); immunostaining (LB and AT), data analysis and data interpretation (LB, AT, JS-H, RL, AH, and GK); contribution of clinical data and material (KB, KO, AN, and AW); critical revising and final approvement of the manuscript (all authors); agreement to be accountable for all aspects of the work in ensuring that questions related to the accuracy or integrity of any part of the work are appropriately investigated and resolved (all authors).

## Conflict of Interest Statement

The authors declare that the research was conducted in the absence of any commercial or financial relationships that could be construed as a potential conflict of interest. The Associate Editor Inti Zlobec declares that, despite having collaborated with author Rupert Langer, the review process was handled objectively and no conflict of interest exists.

## Supplementary Material

The Supplementary Material for this article can be found online at http://journal.frontiersin.org/article/10.3389/fonc.2015.00094/abstract
