# Supplementary material for: Clinical Significance of NOTCH1 and NOTCH2 Expression in Gastric Carcinomas: An Immunohistochemical Study
Source: Front Oncol. 2015 Apr 22;5:94. doi: 10.3389/fonc.2015.00094 (PMC4406075; doi:10.3389/fonc.2015.00094)
Supplement: Supplementary file 1 [file Datasheet_1.PDF]

Supplementary Material

Supplementary Materials and Methods

Immunohistochemistry

Two-micron sections of FFPE tissue were deparaffinised and subjected to heat-induced antigen retrieval in 10 mM citric acid buffer, pH 6. The endogenous peroxidase activity was blocked by incubation with 3 % H_2O_2 for 10 min. Nonspecific antibody binding was blocked in 5 % normal goat serum for 30 min prior to incubation with the primary antibody, undiluted for NOTCH1 and in a 1:30 dilution for NOTCH2, for 1 h at room temperature. A secondary biotinylated rabbit anti-rat antibody (E0468, DAKO, Glostrup, DK, dilution 1:100) was applied for 30 min at room temperature. Streptavidin-peroxidase-based detection was performed with the Dako REAL^{TM} Detection system Peroxidase/DAB+ kit (K5001, DAKO, Glostrup, DK). Serum and antibodies were diluted in Dako REAL^{TM} Antibody Diluent (S2022, DAKO, Glostrup, DK). Tumor staining was classified as evaluable, if at least 50 tumor cells were visible in each biopsy or tissue core. Whole sections of NOTCH1 and NOTCH2 expressing tumors were included as positive controls in each reaction. Additional sections of the same cases were treated without primary antibody to serve as negative controls."

Cell Culture and Knockdown

The GC cell line MKN28 was maintained in RPMI1640 cell culture medium supplemented with 10 % foetal calf serum (FCS), 2 mM glutamine, 100 U/ml penicillin and 1 mg/ml streptomycin. *NOTCH1*- or *NOTCH2*-expression was knocked down by stable transfection of the shRNA constructs TRCN0000003359 (*NOTCH1*), TRCN0000003362 (*NOTCH1*), TRCN0000004894 (NOTCH2) or TRCN0000004895 (NOTCH2) into MKN28 cells. A scrambled non-specific shRNA construct (SHC002) was used to generate a control cell line. All of the constructs were purchased as inserts in pLKO.1puro plasmids from Sigma Aldrich (Hamburg, D). Lentiviral particles were produced in HEK293T and were transiently transfected with 4 µg of vector DNA and 4 µg each of the packaging vectors pMD2.G and psPAX2 (Addgene, Cambridge, MA, USA) using 12 µl of TurboFect (Thermo Scientific, Schwerte, D) in 600 ml of OptiMEM (Gibco, Darmstadt, D). The transfection reagent was exchanged with DMEM medium with 10 % FCS after 8 h. The lentiviral supernatant was harvested every 12 h, mixed in equal parts with RPMI and transferred to MKN28 cells under addition of 8 µg/ml Polybrene (Santa Cruz, Santa Cruz, CA, USA) to transfect the cells. After 3 cycles of transfection, bulk selection of the transfected cells was performed by addition of 2,5 µg/ml Puromycin (Sigma Aldrich, Hamburg, D) to the medium for 10 days, followed by continuous treatment with 1 µg/ml Puromycin. Knockdown efficiency was confirmed by qPCR for *NOTCH1* and *NOTCH2* in regular intervals (data not shown).

Western Blotting

The specificity of the anti-NOTCH1 and anti-NOTCH2 antibodies bTAN20-s and C651.6DbHN was confirmed by western blotting, using total protein from the GC cell line MKN28 and *NOTCH1* and *NOTCH2*-knockdown cell lines derived from MKN28 (Fig. S1 and supplementary material). T-Per buffer (Pierce, Rockford, MD, USA) containing protease inhibitors (Roche Diagnostics, Mannheim, D) was used to lyse $1.5x10^6$ cells. Fifty micrograms of protein was resolved by 5-10 % SDS-PAGE and was blotted to a nitrocellulose

membrane. The membrane was blocked with 5 % dry milk in TBST and was incubated with the primary anti-NOTCH2 antibody (dilution 1:250) or anti-NOTCH1 antibody (dilution 1:100) overnight at 4 °C, followed by an incubation with an HRP-conjugated goat anti-rat F(ab')$_2$ fragment (dilution 1:5000, NA9350, GE Healthcare, Freiburg, D). Signal detection was performed with ChemiGlow West chemiluminescence substrate and the FluorChem SP imaging system (Protein Simple, Santa Clara, CA, USA). Detection of the reference protein β-actin was performed with the same protocol, using a monoclonal primary mouse-anti-β-actin antibody (dilution 1:5000; AC-15, Sigma Aldrich, Hamburg, D) and a secondary HRP-conjugated sheep anti-mouse antibody (dilution 1:10000; NA931, GE Healthcare, Freiburg, D).

Supplementary Figure Legend

Figure S1: Analysis of anti-NOTCH1 and anti-NOTCH2-antibody specificity by western blotting of protein lysates from the GC cell line MKN28 with or without NOTCH1/NOTCH2 knockdown.

A) Western blot with NOTCH1-specific signals at approximately 300 kDa, representing the full-size protein (FS) and a double signal near 120 kDa, representing the activated cleaved forms NEXT (NOTCH extracellular truncation) and NICD (NOTCH intracellular domain); detected using monoclonal anti-NOTCH1 antibody bTAN20-s. **B**) Decreased NOTCH1 signals (FS and NEXT/NICD) in *NOTCH1*-knockdown cell lines compared to controls but not in *NOTCH2*-knockdown cell lines; detected using monoclonal anti-NOTCH1 antibody bTAN20-s **C**) Western blot with NOTCH2-specific signals for NOTCH2-FS near 300 kDa and a double signal near 120 kDa, representing NOTCH2-NEXT and -NICD; detected using monoclonal anti-NOTCH2 antibody C651.6DbHN. **D**) Substantial decrease of NOTCH2 signals (FS and NEXT/NICD) in *NOTCH2*-knockdown cell lines compared to controls but not in *NOTCH1*-knockdown cell lines; detected by monoclonal anti-NOTCH2 antibody C651.6DbHN.

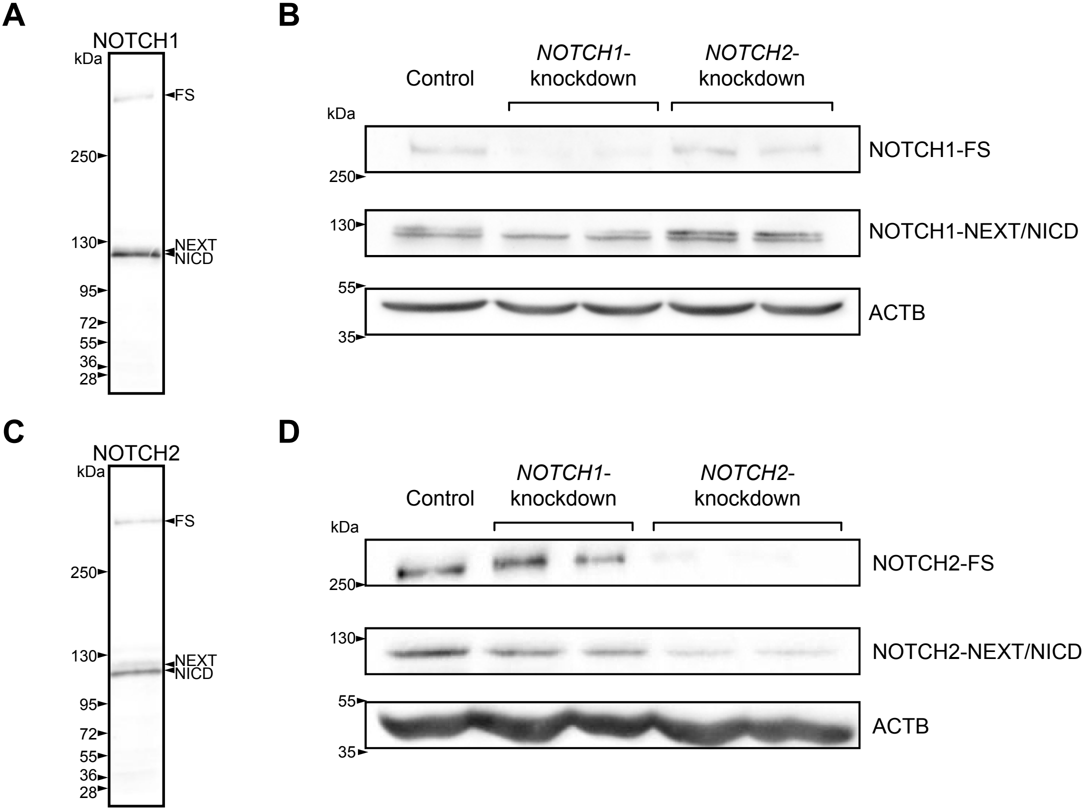
A
NOTCH1
kDa
FS
250
130
NEXT
NICD
95
72
55
36
28
B
Control
NOTCH1-
knockdown
NOTCH2-
knockdown
kDa
NOTCH1-FS
250
130
NOTCH1-NEXT/NICD
55
ACTB
35
C
NOTCH2
kDa
FS
250
130
NEXT
NICD
95
72
55
36
28
D
Control
NOTCH1-
knockdown
NOTCH2-
knockdown
kDa
NOTCH2-FS
250
130
NOTCH2-NEXT/NICD
55
ACTB
35
